# Experiences of gender-based violence among women in sub-Saharan Africa: identifying evidence for intervention and public health priorities

**DOI:** 10.3389/fpubh.2025.1492755

**Published:** 2025-02-12

**Authors:** Lucky Norah Katende-Kyenda, Judith I. Ani

**Affiliations:** Faculty of Medicine and Health Sciences, Walter Sisulu University, Mthatha, South Africa

**Keywords:** gender-based violence (GBV), public health, sub-Saharan Africa, women, sustainable development goals

## Abstract

**Background:**

Gender-based violence (GBV) poses a significant global threat to human rights, public health and attaining the Sustainable Development Goals. There is a growing emphasis on researching to identify issues and understand the experiences of women relative to GBV. The increasing demand for policymakers and public health practitioners to adopt evidence-based approaches in addressing GBV highlights the need for research prioritization on experiences of GBV among women in resource-limited settings such as sub-Saharan Africa. This paper explored GBV among women in 25 sub-Saharan African (SSA) countries to identify and present key intervention priority areas for addressing GBV in these settings.

**Methods:**

The study involved a cross-sectional analysis of a nationally representative dataset from the Demographic and Health Survey of 25 SSA African countries. Data was analyzed descriptively and inferentially using the Pearson chi-square (X2) at a *p* < 0.05.

**Results:**

Findings revealed that women aged 25–29, living in rural areas and with primary education were at a significant risk of experiencing GBV across 25 SSA countries. A notable 44.94% of women reported experiencing at least one form of GBV, with the prevalence varying by country. Women in Comoros had the lowest reported prevalence (10.76%), while Sierra Leone women had the highest (60.27%), followed by Uganda (56.92%). Emotional and physical violence were most prevalent in Sierra Leone, while sexual violence was most common in Burundi and the Democratic Republic of Congo.

**Discussion:**

This study highlights the urgent need for targeted interventions for younger women in rural areas and those with limited education. By prioritizing evidence-based approaches, stakeholders can develop more effective, sustainable, and impactful programs to reduce GBV and improve the well-being of women across the SSA region.

## Introduction

One of the primary objectives of the Sustainable Development Goals (SDGs) is to ensure equal opportunities for all genders and address disparities across various sectors (1). Unfortunately, the achievement of these goals faces consistent challenges due to the persistent rise of gender-based violence (GBV). Globally, gender-based violence (GBV) is a significant public health issue and a violation of human rights, with a particularly severe impact in developing regions, including sub-Saharan Africa (SSA) (2, 3). The experience of GBV endangers the lives of countless women and restricts their autonomy, making it difficult for them to prioritize health concerns for themselves and their children (4). GBV are multifaceted and encompasses various forms of violence rooted in unequal power relations, gender norms and societal expectations. It affects individuals based on their gender or gender identity but disproportionately affects women and girls. Forms of GBV includes physical, sexual, and emotional, economic or financial, cultural or harmful traditional practices, structural, digital or online, stalking, reproductive, human trafficking and exploitation (5, 6). Physical violence involves the deliberate use of force or power, whether threatened or actual, that can result in harm, injury, or even death. Sexual violence refers to any forced or coerced sexual act imposed on someone against their will. Emotional violence includes actions, threats, or coercive tactics designed to inflict trauma or harm an individual’s self-esteem, identity, or personal development. Economic or financial violence refers to controlling or restricting someone’s access to financial resources, employment or opportunities to sustain themselves. These include actions like withholding money and preventing someone from working. Cultural or harmful traditional practices represent deeply entrenched behaviors and rituals that often violate fundamental human rights and dignity, frequently under the guise of preserving cultural or religious norms. These practices disproportionately affect women and girls, reinforcing gender inequality and perpetuating cycles of violence. Female genital mutilation (FGM), for instance, is a severe violation of bodily autonomy and a cause of lifelong physical and psychological harm. Similarly, child marriage and forced marriage strip individuals of their agency, exposing them to abuse and limiting their opportunities. Other manifestations include dowry-related violence, where women face harm or death due to unmet financial demands, and widow inheritance, which forces women into unwanted unions after their husband’s death.

Structural violence operates on a systemic level, embedding gender-based discrimination within the fabric of societal institutions and norms. It manifests through inequalities in access to education, healthcare, and justice, as well as the underrepresentation of marginalized groups in political and economic spheres. Structural violence is insidious because it perpetuates itself through established systems, making it harder to challenge and dismantle. For instance, women and girls in many regions face barriers to education, which then limits their opportunities and exacerbates cycles of poverty and disempowerment. Digital or online violence is an emerging and increasingly pervasive form of abuse, facilitated by advancements in technology and the ubiquity of social media platforms. This form of violence often includes cyberstalking, where perpetrators monitor or harass victims online, creating a constant state of fear and insecurity. Revenge pornography, the non-consensual sharing of intimate images, violates privacy and can have devastating personal and professional consequences. Online harassment, doxxing (the public release of personal information), and other forms of digital abuse illustrate how technology can be weaponized to inflict harm.

Stalking is another form of gender-based violence that involves persistent and unwanted attention or surveillance, causing immense fear and distress to victims. This behavior can escalate into more severe forms of violence if left unchecked. Stalkers may follow their victims, repeatedly send threatening or harassing messages, or appear uninvited at their homes, workplaces, or other personal spaces. The invasive nature of stalking undermines victims’ sense of safety and autonomy. Reproductive violence infringes on individuals’ reproductive rights and autonomy, often with long-lasting consequences. Acts such as forced sterilization and forced abortion are stark violations of bodily integrity, frequently carried out without consent or under coercion. Denial of access to contraception and coerced pregnancies further limit individuals’ ability to make decisions about their reproductive health and family planning, reinforcing unequal power dynamics.

Human trafficking and exploitation represent some of the most extreme forms of gender-based violence, stripping individuals of their freedom and exploiting them for labor, sexual purposes, or other ends. Victims, many of whom are women and children, are subjected to unimaginable abuse and degradation. Sex trafficking forces individuals into commercial sexual exploitation, while forced labor and organ trafficking violate their fundamental rights and reduce them to commodities in an illicit global trade. These highlight the pervasive nature of the different forms of GBV. However, this study focuses on physical, emotional and sexual violence.

Globally, it is estimated that one in three women will experience some form of GBV during their lifetime, often beginning as early as age 15 (7). Additionally, a World Health Organization (WHO) study on violence and women’s health, conducted across 10 countries, revealed that 13–62% of women had experienced physical violence at some point in their lives (8). These statistics underscore the widespread and pervasive nature of GBV, affecting women across different cultural and socioeconomic contexts.

In SSA where low socioeconomic status, limited access to education, patriarchy and unequal power balances are prevalent, GBV persists. Utilizing nationally representative data from Demographic and Health Surveys (DHS), this study was conducted to gain a deeper understanding of the experiences of GBV among ever-married women in intimate-partner relationships in sub-Saharan Africa (SSA). The focus on SSA is particularly important, as the majority of existing studies on GBV have been concentrated in developed countries, leaving a significant gap in evidence and understanding of the issue within SSA countries (2, 3). Also, the region is known for having one of the highest prevalence rates of GBV among women and girls globally (9), making it a critical focus for this study. This study aims to fill that gap by providing comprehensive insights into the experiences of various kinds of GBV in this region, thereby, contributing to more informed and effective interventions tailored to the unique challenges faced by women in SSA.

The World Health Organization (WHO), through a comprehensive multi-country study, has established a strong link between GBV and a wide range of adverse health outcomes, including severe mental health issues and an increased risk of suicidal behavior (10–14). These studies highlight that a history of physical or sexual abuse is associated with numerous costly health conditions that place a significant burden on healthcare systems. Additionally, survivors of GBV are more likely to suffer from serious mental health problems, including depression, anxiety, post-traumatic stress disorder (PTSD), and a heightened risk of suicide. The pervasive impact of GBV on both physical and mental health underscores the urgent need for targeted interventions to address these challenges, particularly in regions where healthcare resources are already strained.

Given this established link between GBV and numerous severe health conditions, the need for focused research on GBV experiences among women in SSA becomes even more critical. SSA is a region characterized by significant socioeconomic challenges, limited healthcare resources, and socio-cultural issues that affect mostly women. Despite the pervasiveness of GBV and its detrimental effects, it has been acknowledged that global evidence base has been a slow progress on GBV (9). This paucity limits the development of effective interventions tailored to the unique cultural and social dynamics of SSA. This study is, therefore, justified by the pressing need to understand the specific experiences of GBV within SSA, which is essential for identifying intervention priorities that are both contextually relevant and impactful. Without robust, localized data, interventions may fail to address the root causes of GBV or effectively mitigate its widespread health impacts. By focusing on the experiences of GBV in SSA, this research aims to fill the critical gap in evidence, thereby enabling the design of targeted, evidence-based strategies that can more effectively reduce the burden of GBV and its associated health consequences in the region. Operationally, this study interchangeably and synonymously used GBV, intimate partner violence (IPV) and domestic violence. IPV refers to violence experienced in an intimate relationship. While GBV is more encompassing, IPV is one of the forms of GBV, although IPV, GBV and domestic violence are sometimes used interchangeably.

## Methods

To assess the experiences of GBV among women in SSA, this study conducted a cross-sectional analysis of Demographic and Health Survey (DHS) data from 25 sub-Saharan African countries. The surveys selected for this analysis, conducted between 2012 and 2022, represent the most recent data available for each country at the time of the research (refer to Table 1). The DHS, conducted in over 80 countries, primarily low-and middle-income nations, collects nationally representative data using standardized and comparable questions on various social and health issues. This study specifically focused on ever-married women aged 15–49 who participated in the domestic violence and other relevant modules. By utilizing the most recent and comprehensive dataset, this research provides a analysis of GBV experiences in the region. The datasets used for this study are publicly accessible at: https://dhsprogram.com/data/available-datasets.cfm. The countries included in the sample were Angola, Benin, Burundi, Cameroon, Chad, Comoros, Democratic Republic of Congo, Gabon, The Gambia, Kenya, Liberia, Madagascar, Malawi, Mali, Mauritania, Namibia, Nigeria, Rwanda, Sierra Leone, South Africa, Tanzania, Togo, Uganda, Zambia, and Zimbabwe.

**Table 1:** Sociodemographic characteristics of respondents.

Variable	Frequency (*N* = 122,477)	Percentage (100%)
Age group		
15–19	6,956	5.68
20–24	21,231	17.33
25–29	27,618	22.55
30–34	24,440	19.95
35–39	19,348	15.80
40–44	13,392	10.93
45–49	9,492	7.75
Residence		
Urban	45,755	37.36
Rural	76,722	62.64
Educational attainment		
No education	33,556	27.40
Primary	46,292	37.80
Secondary	35,418	28.92
Tertiary	7,211	5.89
Media exposure		
Not Exposed	104,616	85.42
Exposed	17,861	14.58
Wealth Index		
Poorest	24,282	19.83
Poorer	24,400	19.92
Middle	24,370	19.90
Richer	25,297	20.65
Richest	24,128	19.70
Parity		
None	7,366	6.01
1–4	79,681	65.06
5+	35,430	28.93

To understand and assess GBV experiences among women in SSA, the study utilized measures from the domestic violence module of the DHS. This module captures reports of physical, emotional, and sexual violence, collectively referred to as GBV. Physical violence was defined as instances where husbands or partners hit, slapped, kicked, or inflicted any form of physical harm on the women. Emotional violence included experiences of humiliation, threats, or insults. Sexual violence was defined as being forced to have intercourse against the woman’s will or without her consent. Women who experienced any or all of these forms of violence were considered to have experienced GBV.

The analysis of GBV was conducted in two stages: first, as a composite variable encompassing all forms of violence, and second, by examining each type of violence separately. Frequency and percentage distributions were generated for each form of GBV. Additionally, each type of GBV was disaggregated by explanatory variables to identify which demographic groups were most affected. Variables were subjected to Pearson chi-square (*X*^2^) test at a *p* < 0.05. Ethical approval for this study was obtained from the Ethics Committee of ICF Macro International, Inc., Calverton, Maryland, as well as from the National Ethics Committee of each participating country. Although the dataset is publicly available, formal permission to use the data was secured. Comprehensive details regarding the data and adherence to ethical standards can be found at Ethical Standards and Data Use^1^.

## Results

### Sociodemographic characteristics of respondents

Table 1 presents the sociodemographic characteristics of the respondents across the 25 sub-Saharan African countries included in the study. The majority of respondents (22.55%) were aged between 25 and 29 years, highlighting the high reproductive age and youthful population that is typical of sub-Saharan Africa. Nearly two-thirds (62.64%) of the respondents resided in rural areas, and a significant proportion (37.80%) had only attained primary education. Additionally, more than four in every five respondents (85.42%) had no media exposure, all of which are indicators of low socioeconomic status prevalent in the region.

### Experiences of GBV by countries in SSA

Over four in 10 women (44.94%) across the sampled countries reported experiencing at least one form of gender-based violence (GBV), as shown in Table 2. Women in Comoros had the lowest reported experiences of GBV, with 10.76% indicating they had encountered any form of violence. In contrast, women in Sierra Leone had the highest prevalence, with 60.27% reporting experiences of GBV, followed closely by Uganda at 56.92%. Sierra Leone also had the highest percentage of women who experienced both emotional (46.26%) and physical (48.13%) violence. Meanwhile, sexual violence was most prevalent among women in Burundi (25.85%) and the Democratic Republic of Congo (25.38%). These findings highlight significant variations in the prevalence and types of GBV across different countries in sub-Saharan Africa.

**Table 2:** Percentage distribution of experiences of GBV by countries n SSA.

Countries	Ever experienced GBV	Ever experienced emotional violence	Ever experienced physical violence	Ever experienced sexual violence
Angola	3,005 (40.22%)	1876 (25.11%)	2,428 (32.49%)	590 (7.90%)
Benin	1792 (40.98%)	1,560 (35.67%)	805 (18.41%)	359 (8.21%)
Burundi	3,511 (50.48%)	1781 (25.61%)	2,722 (39.14%)	1798 (25.85%)
Cameroon	1963 (43.66%)	1,260 (28.02%)	1,521 (33.83%)	463 (10.30%)
Chad	1,093 (29.80%)	735 (20.04%)	826 (22.52%)	324 (8.83%)
Comoros	269 (10.76%)	205 (8.20%)	140 (5.60%)	42 (1.68%)
Congo DR	3,130 (56.39%)	2026 (36.50%)	2,552 (45.97%)	1,409 (25.38%)
Gabon	1,231 (44.34%)	896 (32.38%)	950 (34.22%)	294 (10.59%)
The Gambia	775 (40.62%)	501 (26.26%)	583 (30.56%)	118 (6.18%)
Kenya	5,534 (44.93%)	4,263 (34.61%)	4,021 (32.64%)	1,255 (10.19%)
Liberia	1,246 (54.87%)	952 (41.92%)	1,031 (45.40%)	170 (7.49%)
Madagascar	2,277 (39.25%)	1855 (31.97%)	1,289 (22.22%)	606 (10.44%)
Malawi	2,181 (41.63%)	1,485 (28.35%)	1,319 (25.18%)	999 (19.07%)
Mali	1,458 (44.06%)	1,144 (34.57%)	1,067 (32.25%)	336 (10.15%)
Mauritania	595 (18.29%)	493 (15.15%)	177 (5.44%)	199 (6.12%)
Namibia	477 (34.27%)	351 (25.22%)	345 (24.78%)	102 (7.38%)
Nigeria	3,281 (38.04%)	2,855 (33.10%)	1779 (20.62%)	661 (7.66%)
Rwanda	867 (46.41%)	650 (34.80%)	687 (36.78%)	289 (15.47%)
Sierra Leone	2,382 (60.27%)	1828 (46.26%)	1902 (48.13%)	304 (7.69%)
South Africa	546 (24.54%)	424 (19.06%)	340 (15.28%)	84 (3.78%)
Tanzania	3,346 (45.49%)	2,376 (32.30%)	2,659 (36.15%)	910 (12.37%)
Togo	1938 (37.53%)	1,624 (31.45%)	1,125 (21.79%)	413 (8.00%)
Uganda	4,143 (56.92%)	3,039 (41.76%)	3,024 (41.55%)	1,657 (22.77%)
Zambia	3,363 (47.53%)	2,173 (30.71%)	2,631 (37.19%)	1,046 (14.78%)
Zimbabwe	2,476 (44.94%)	1729 (31.38%)	1,677 (30.44%)	648 (11.76%)
Total	52,879 (43.23%)	38,081 (31.13%)	37,600 (30.74%)	15,076 (12.33%)

### Experiences of GBV in SSA by respondents’ characteristics

As detailed in Table 3, GBV is notably prevalent among women aged 25–29 years, with this age group showing the highest incidence of both physical and sexual violence. Similarly, women aged 30–34 years also report significant experiences of both emotional and physical violence, highlighting a critical period where women in these age groups are particularly vulnerable.

**Table 3:** Percentage distribution of GBV experienced by respondents’ characteristics.

Variable	GBV	Emotional violence	Physical violence	Sexual violence	*p*-value
Age group					0.000
15–19	4.79	4.40	4.55	5.59	
20–24	17.03	16.27	17.06	18.10	
25–29	22.56	22.47	22.55	22.11	
30–34	20.35	20.54	20.46	20.67	
35–39	16.16	16.37	16.20	15.78	
40–44	11.08	11.59	11.20	10.33	
45–49	8.04	8.36	7.99	7.42	
Residence					0.000
Urban	33.0	33.43	33.01	29.71	
Rural	67.0	66.57	66.99	70.29	
Educational attainment					0.000
No education	28.16	28.35	27.91	26.17	
Primary	41.53	40.90	43.06	46.92	
Secondary	26.83	27.03	26.13	24.45	
Tertiary	3.48	3.71	2.90	2.46	
Media exposure					0.000
Not exposed	88.96	89.01	89.24	91.56	
Exposed	11.04	10.99	10.76	8.44	
Wealth index					0.000
Poorest	24.00	23.99	24.91	24.29	
Poorer	21.38	21.30	21.80	22.39	
Middle	20.70	20.67	20.60	20.71	
Richer	19.22	19.16	18.87	19.24	
Richest	14.70	14.88	13.82	13.37	
Parity					0.000
None	4.22	4.03	3.70	4.32	
1–4	63.17	63.12	62.71	62.25	
5+	32.61	32.85	33.59	33.43	

Across the board, rural dwellers experienced higher rates of GBV, including emotional, physical, and sexual violence, compared to their urban counterparts. This trend is consistent with the higher incidence of GBV among women with only primary education or no formal education, who are also more likely to be emotionally and physically abused. These women, along with those who have no exposure to mass media, are disproportionately affected by all forms of violence, emphasizing the role of education and access to information in mitigating GBV.

Women categorized as the poorest report the highest prevalence of all forms of GBV, including emotional, physical, and sexual violence, indicating a strong correlation between socioeconomic status and vulnerability to violence. Additionally, women with one to four children report higher experiences of GBV across all categories, compared to those with no children or more than five children.

The findings indicate that younger women, particularly those in rural areas, with lower educational attainment, limited media exposure, and poorer socioeconomic status, are at the highest risk of experiencing GBV in its various forms.

## Discussion

This paper explored the experiences of GBV among women in SSA using data from the multi-country analysis of the Demographic and Health Survey (DHS) to identify evidence for intervention and public health priorities. Data reveals significant variability in the prevalence of GBV across different countries in SSA, indicating that GBV is not uniformly experienced across the region. For instance, while countries like Sierra Leone and Uganda reported alarmingly high rates of GBV, with over half of the women experiencing violence, others such as Comoros showed considerably lower rates. These disparities in GBV cases across countries in SSA can be attributed to several contextual factors that help explain the varying prevalence rates observed. Cultural norms around gender roles and violence play a critical role in shaping these disparities. In countries where patriarchal values are deeply entrenched, gender inequality tends to be more widespread, contributing to higher rates of GBV. Additionally, harmful cultural practices such as female genital mutilation (FGM) or early marriage, still prevalent in some of these countries like Sierra Leone (15), exacerbate the vulnerability of women to both physical and sexual violence. These practices often perpetuate gender-based discrimination, limiting women’s autonomy and increasing their risk of abuse. Another key factor contributing to the disparities in GBV is the experience of conflict and post-conflict situations (16–18). Countries such as Sierra Leone, Liberia, and Uganda, which have experienced civil wars or prolonged conflicts in recent decades, reported higher rates of GBV, particularly sexual violence. The breakdown of social structures during conflict, along with widespread displacement, leads to heightened vulnerability as women become targets of exploitation.

Also, economic factors, socioeconomic disparities and poverty are strongly linked to the prevalence of GBV in these countries. Women from impoverished backgrounds or those with limited access to education and employment opportunities are at an increased risk of experiencing violence. In Mali where poverty is rife, GBV was prevalent. This finding was corroborated by scholars (19) who found that in countries in Mali, GBV was high and associated with poverty. The Gender Equality Index Report, which evaluated key areas such as reproductive health, employment, and empowerment, revealed that 27 of the 30 countries with the most pronounced gender inequality are located in Africa (20). This underscores a widespread and deep-rooted challenge in achieving gender equality across the continent, indicating a critical need for targeted interventions and comprehensive policy reforms to address these disparities. Economic dependency, combined with a lack of financial independence and limited access to resources like education or healthcare, can trap women in abusive situations. Women in these circumstances often feel they cannot escape violent relationships because they lack the means to support themselves or seek assistance. Thus, addressing poverty and economic inequality is a crucial step in reducing vulnerability to GBV.

Emotional violence (31.13%) emerged as a pervasive form of GBV, with a significant portion of women across the region reporting experiences of this type of abuse. This was followed by physical violence (30.74%) and sexual violence (12.33%) as found in other studies (16). The high prevalence of emotional violence points to a critical but often overlooked dimension of GBV that has profound implications for women’s mental health and well-being. This finding highlights the necessity for comprehensive GBV interventions that address all forms of violence, including emotional abuse. Public health strategies and social services must be equipped to recognize and respond to the psychological impacts of GBV, which are often as debilitating as physical injuries. Physical and sexual violence also remained prominent forms of GBV, with physical violence being particularly widespread. The data shows that in countries like the Democratic Republic of Congo and Uganda, physical violence was experienced by a large proportion of women, underscoring the need for interventions that directly address this form of violence. Meanwhile, the significant rates of sexual violence in countries such as Burundi and the Democratic Republic of Congo demand urgent attention. These findings, therefore, suggest that GBV prevention programs need to include comprehensive measures that address all these forms of violence, with particular emphasis on the legal and medical support systems required to protect and support survivors. It also suggests that GBV interventions must be tailored to the specific sociocultural and economic contexts of each country. National and regional policies need to account for these differences, ensuring that interventions are culturally sensitive and appropriately targeted to address the unique drivers of GBV in each setting.

Moreover, the overall high prevalence of GBV across sub-Saharan Africa underscores the urgent need for robust policy responses. The data calls for the integration of GBV prevention into broader efforts aimed at improving women’s socioeconomic status, as poverty and limited access to education and healthcare exacerbate the risk and consequences of GBV. This indicates that GBV cannot be effectively addressed in isolation but must be part of a holistic strategy that includes legal reforms, economic empowerment, and improved access to essential services.

The demographic characteristics revealed a high reproductive age and youthful population, with a significant portion of women living in low socioeconomic conditions, which is typical of SSA. The findings indicated that more than four in 10 women across the sampled countries reported experiencing at least one form of GBV, with significant variations in prevalence and types of violence across different SSA countries. This aligns with a report (9) which stated that over one in four females have suffered at least one type of violence. However, the actual figures might be higher, as the World Health Organization (21) suggested that many GBV cases go unreported due to fear or coercion.

The GBV was found to be particularly high among women aged 25–29 years, with physical and sexual violence being the most prevalent forms. Cultural beliefs and traditions in many African societies strongly reinforce male dominance, especially in sexual relationships and within marriage (22). These entrenched cultural norms contribute to the systemic nature of GBV and pose significant barriers to gender equality. The findings of this study indicate that women aged 25–29 reported the highest prevalence of gender-based violence (GBV), particularly in the forms of physical and sexual violence. Several factors contribute to the higher rates of GBV within this age group. One key factor is the reproductive and marital pressures that women in this age range often face. Women aged 25–29 are typically at the peak of their reproductive years, and many in this group may experience significant societal expectations regarding marriage, childbearing, and fulfilling traditional roles within the family. These pressures, combined with limited economic and emotional independence, create vulnerabilities to violence. This age group often overlaps with those in early marriages, which are particularly prevalent in sub-Saharan Africa, and young mothers within these relationships are more likely to experience abuse due to the power imbalances that can characterize these dynamics.

Additionally, this age range represents a critical transition from adolescence to adulthood, during which many women may still lack the life experience or the social resources to navigate violent situations. In many cases, women in this group have limited access to education or employment opportunities, which further compounds their vulnerability. The absence of financial independence and the inability to secure alternate means of support make it more difficult for these women to leave abusive situations. This highlights the increased susceptibility of younger women to violence, as they may not have the necessary resources, both social and financial, to escape abusive relationships.

Economic vulnerability and dependency are also significant risk factors contributing to the high rates of GBV in this age group. Many women aged 25–29, particularly in rural areas or low-income settings, may be economically dependent on their partners, which can entrench their involvement in violent relationships. The lack of financial independence makes it much more difficult for women to leave abusive situations, as they may fear economic hardship or the inability to provide for themselves or their children. This is reflected in the higher rates of GBV among women with lower educational attainment or those in the poorest wealth index categories, emphasizing the strong connection between economic insecurity and vulnerability to violence. Therefore, any effective intervention strategy must address these cultural attitudes to create meaningful change.

Geographical isolation and rurality further compounded the vulnerability of women. As revealed in the study, women in rural areas experienced higher rates of GBV compared to their urban counterparts. This disparity may be attributed to power imbalances, lower levels of education, and reduced socioeconomic empowerment in rural areas. The cultural context in these areas often places men in dominant positions, which can perpetuate GBV. Also, women in rural areas often have limited access to communication platforms such as television, radio, or the internet. This isolation exacerbates their susceptibility to GBV, as rural regions are often characterized by deeply entrenched traditional gender norms that perpetuate violence. The study revealed a higher prevalence of GBV among rural women, suggesting that targeted interventions, such as increasing media access and outreach efforts in rural areas, could play a significant role in mitigating GBV. Additionally, the absence of media access acts as a broader barrier to information. Without access to critical information that can help them protect themselves or access resources, women in marginalized or economically disadvantaged communities remain trapped in environments where GBV persists unchecked. This finding highlights the importance of implementing gender-sensitive education and advocacy programs that challenge harmful stereotypes and promote equality within relationships.

Generally, nearly 63% of the African population lives in remote rural areas where access to basic amenities and services is severely limited (23). This geographic isolation exacerbates the vulnerability of rural populations to GBV, as limited access to resources such as healthcare, legal support, and economic opportunities can prevent victims from seeking help or escaping abusive situations. Economically empowered women are more likely to resist GBV and take prompt action against it, but many rural women lack such empowerment. Additionally, rural communities are often isolated from the influence of central governments, media and the enforcement of laws designed to prevent GBV, making it difficult to implement protective measures effectively. This lack of government presence and law enforcement in rural areas (24) means that GBV frequently goes unchecked, emphasizing the need for localized approaches that empower communities and improve access to essential legal and social services.

The lack of media exposure among women was strongly correlated with higher rates of gender-based violence (GBV). Corroborating other studies (25), women without media exposure (85.42%) reported the highest prevalence of all forms of GBV—emotional, physical, and sexual violence. Notably, women without access to media often remain unaware of their legal rights, available protections, and support services. This lack of awareness impedes their ability to recognize abuse, report incidents, or seek help. Media campaigns serve as vital tools in raising awareness, educating women about their rights, and providing information on avenues for support. The absence of such exposure leaves many women uninformed and more susceptible to violence.

Educational attainment is another significant variable influencing GBV prevalence (26). Women with no formal education or only primary education (65.2%) reported significantly higher rates of GBV compared to those with secondary or tertiary education. Education serves as a protective factor, providing women with the skills, opportunities, and networks to achieve financial independence and seek help when necessary. In contrast, a lack of education limits employment opportunities and fosters economic dependency, leaving women more vulnerable to violence.

The study also highlighted the role of economic vulnerability, as demonstrated by the wealth index. Women in the poorest wealth quintiles (24.00%) reported the highest rates of GBV, underscoring the link between financial insecurity and vulnerability to abuse. Economic dependency often traps women in abusive relationships, as they lack the resources needed to leave or seek protection. This position has also been justified by other findings (27–29).

The findings also revealed a strong association between GBV and parity. Women with 1–4 children reported higher rates of GBV compared to those with no children. This pattern may reflect the increased emotional and physical burden of caregiving in environments of abuse, as well as the challenges of raising children with limited resources or support. Women in this category may also feel more compelled to remain in violent relationships to ensure the well-being of their children, further exposing them to continued violence.

The findings of the study have significant implications for establishing intervention priorities and shaping policies to effectively address gender-based violence (GBV) in sub-Saharan Africa. The high prevalence of GBV among younger women, particularly those aged 25–29, underscores the need for targeted interventions focused on this demographic. To address this issue, programs should prioritize early intervention and education about GBV, particularly for young women. By focusing on this age group and providing comprehensive support and education, it is possible to play a crucial role in reducing instances of violence and improving overall outcomes for women in the region.

Given that data suggests that women in rural areas are at heightened risk of experiencing GBV, it highlights the need for targeted interventions that focus on these vulnerable groups. Rural women, who often have limited access to resources and support, may benefit from programs that enhance their economic empowerment and provide accessible healthcare and legal services. Addressing these specific vulnerabilities is crucial for reducing GBV and promoting gender equality across the region. There is need to enact specific laws that criminalize and prohibit GBV. This legal shortfall demonstrates a critical gap in the protection of women and other vulnerable groups, underscoring the need for more comprehensive legal frameworks and stronger political will to combat GBV across the continent. To effectively combat gender-based violence (GBV) in SSA, it is crucial for governments to prioritize the development and implementation of comprehensive and context-specific policies. These policies should be designed to address the unique cultural, social, and economic challenges faced by communities in the region. By focusing on both immediate and long-term prevention strategies, these policies can lay the foundation for meaningful change.

Strengthening legal frameworks is essential to ensure that GBV is not only recognized but also effectively addressed. Legal provisions must go beyond criminalization to include social support systems that provide survivors with access to education, economic opportunities, and comprehensive health services. These frameworks should be enforced consistently across SSA, ensuring that all individuals, particularly women and vulnerable populations, are protected. Efforts to address GBV must also be closely aligned with the Sustainable Development Goals (SDGs), particularly the target of eradicating intimate partner violence by 2030. This alignment will require coordinated actions at the national and regional levels, with a focus on integrating GBV prevention into broader development agendas. Governments and stakeholders must develop and implement immediate action plans that respond to the specific challenges faced by women in SSA, ensuring that interventions are timely, effective, and sustainable. Finally, the evidence presented in this study also highlights the need for continued research on the dynamics of GBV in SSA. By building on the current body of knowledge, stakeholders can develop more effective strategies to prevent GBV and support survivors across the region.

## Study limitations

This study provides valuable data on gender-based violence (GBV) among women aged 15–49 in sub-Saharan Africa (SSA), but several limitations must be acknowledged. A key limitation is its cross-sectional design, which captures data at a single point in time. This prevents understanding of the temporal progression of GBV, including when it began, how it evolves, or its causal relationships with other variables. Underreporting is another concern, as GBV is highly stigmatized in SSA. Cultural norms and societal pressures often discourage disclosure, with women fearing retribution, shame, or protecting family members. Even when incidents are reported, the frequency and severity of violence are likely underrepresented, masking the full scope of the issue.

Additionally, reliance on self-reported data introduces recall bias, especially for traumatic experiences where memories may be fragmented or deliberately suppressed. The study also does not include some predictors of GBV identified in previous research, such as economic stress, mental health, substance abuse, or communication dynamics within relationships. These omissions may limit the comprehensiveness of the findings. Despite these limitations, the study highlights critical gaps in knowledge and emphasizes the need for future research. Longitudinal studies are essential to track the onset, duration, and progression of GBV and to explore a wider range of contributing and protective factors.

## Data Availability

Publicly available datasets were analyzed in this study. This data can be found at: https://dhsprogram.com/data/available-datasets.cfm.

## Footnotes

^1^ http://goo.gl/ny8T6X
